# Exploring the quality of life and its determinants among caregivers of patients with tuberculosis: a cross-sectional study

**DOI:** 10.1186/s12889-025-23854-2

**Published:** 2025-08-19

**Authors:** Esraa Abdellatif Hammouda, Rasha Ashmawy, Mahmoud A. Hassan, Ramy Mohamed Ghazy

**Affiliations:** 1 Medical Research Institute, Alexandria University, Alexandria, Egypt; 2 Clinical Research Department, El-Raml Pediatric Hospital, Ministry of Health and Population, Alexandria, Egypt; 3 Clinical Research Department, Maamora Chest Hospital, Ministry of Health and Population, Alexandria, Egypt; 4 Institute of Graduate Studies and Research, Alexandria University, Alexandria, Egypt; 5 Family and Community Medicine Department, College of Medicine, King Khalid University, Abha, Saudi Arabia; 6 Tropical Health Department, High Institute of Public Health, Alexandria University, Alexandria, Egypt

**Keywords:** Quality of life, Tuberculosis, Caregivers, Burden of disease

## Abstract

**Background:**

Assessing quality of life (QoL) of caregivers of patients with tuberculosis (TB) highlights their unseen sacrifices. This study aimed to address the domains of QoL of TB caregivers and to estimate their possible determinants in Alexandria, Egypt.

**Methods:**

This cross-sectional survey was conducted in the chest clinics and the main chest hospital in Alexandria, Egypt. From May to September 2023, data were collected through structured, face-to-face interviews using the World Health Organization Quality of Life– BREF (WHOQOL-BREF) questionnaire. The findings were compared to those of published results from tuberculosis patients and the general population. Multivariate regression analysis was conducted to identify the key predictors influencing the QoL of TB caregivers.

**Results:**

In total, 149 caregivers participated in the study; 83.9% of them were females, and 76.5% were married. Caregivers showed QoL scores similar to TB patients in most domains (*p* > 0.05), except for the social domain, where they scored significantly lower (39.7 ± 20.2 vs. 50.3 ± 20.6; *p* < 0.001). Compared to the general population, caregivers had significantly lower QoL across all domains. In the physical domain, older age (≥ 65 years) predicted lower QoL (*β* = −16.45, *p* = 0.022), while male gender and the absence of chronic disease were associated with higher scores (*β* = 10.48, *p* = 0.022 and *β* = 15.51, *p* < 0.001, respectively). The psychological domain was positively affected by the absence of chronic disease (*β* = 8.23, *p* = 0.015). For social relations, single and widowed/divorced individuals reported markedly lower QoL than married participants (*β* = −20.96, *p* = 0.003 and *β* = −20.18, *p* < 0.001, respectively). Lastly, in the environmental domain, receiving additional caregiving support predicted improved QoL (*β* = 5.42, *p* = 0.039).

**Conclusion:**

TB significantly impaired the QoL of the caregivers. These findings highlight the need for the targeted interventions to improve their well-being.

**Supplementary Information:**

The online version contains supplementary material available at 10.1186/s12889-025-23854-2.

## Introduction

The World Health Organization (WHO) considers Tuberculosis (TB) as a preventable and usually curable disease caused by the bacillus *Mycobacterium Tuberculosis*. After exposure, the risk of developing active TB disease increases to approximately 5% in the first 2 years, then it decreases. The disease typically affects the lungs and is called pulmonary TB, but it can affect other sites such as the kidney, spine, or brain (extra-pulmonary TB) [1, 2]. 

TB remains the leading cause of death from communicable diseases with 1.3 million people died from TB in 2023 [1]. According to the global report of TB by WHO, the number of people newly diagnosed with TB was 10 million yearly, with a great increase from last year: 7.1 million in 2019, 5.8 million in 2020, to 6.4 million in 2021. The global average incidence of TB is 135 cases per 100,000 people in 2023, most of them in high TB burden countries, with 87.0% of them in low and middle-income countries (LMICs). Five countries account for 56% of the global TB burden: India (26%), Indonesia (10%), China (6.8%), the Philippines (6.8%), and Pakistan (6.3%). Among reported TB cases, approximately 55% were men, 33% were women, and 12% were children aged 0–14 years. The Eastern Mediterranean Region (EMR) has rebounded from the coronaviruse 2019 (COVID-19)-related decline in TB diagnosis, reporting 116 cases per 100,000 population in 2023, of which 6.6% were multidrug-resistant. Egypt has a reduction in TB incidence to 11,000 cases in 2023, with a rate of 9.2 cases per 100,000 population, with 0.6% MDR cases compared to 10 cases/100,000 population in 2021 [3]. 

Treatment phase duration and drug content may differ according to the approved policy of treatment. The United States Centers for Disease Control and Prevention (CDC) stated a 4-month daily dose of the rifapentine-moxifloxacin regimen [4] while the WHO, American Thoracic Society, and Infectious Diseases Society of America (IDSA) stated a guideline for the treatment of TB includes 2 phases started by an intension phase of 2 months of isoniazid, rifampicin, pyrazinamide, and ethambutol, and followed by a continuation phase of 4 months of isoniazid and rifampicin. The Egyptian Ministry of Health and Population (MoHP) has approved the lastest guideline of treatment with a directly observed therapy short-course system (DOTS) [5–7].

Before and throughout treatment, patients with TB have challenges in conducting their tasks, necessitating the assistance of family members. Caregivers have become an important position as part of the healthcare team that handles patients with physicians. Informal care from relatives or family members accounts for most home care. Furthermore, informal caregiving now has a far higher economic value than paid services, especially in Eastern and Islamic communities, due to their social norms and traditions that value providing care by family members and close relatives for the population who need help. This has increased the burden on caregivers and affects their quality of life [8].

QoL is a recent term in the healthcare system used to indicate overall well-being, happiness, and satisfaction with life [9]. The WHO defines QoL as "an individual’s perception of their position in life in the context of the culture and value systems in which they live and in relation to their goals, expectations, standards, and concerns" [10]. Zarit et al. [11], have defined caregiver burden as “the extent to which caregivers perceive that caregiving has had an adverse effect on their emotional, social, financial, physical, and spiritual functioning”. Despite the poor QoL of caregivers is not a clinical diagnosis, caregivers suffer physical burdens such as pain, fatigue, poor self-care, lack of sufficient rest, and sleep disturbance [12]. Psychological burdens like depression, anxiety, feeling guilty, and sadness [13]. Caregivers may also challenge social interactions: stigma and discrimination may lead to social isolation and loss of social support [14]. Financial burden may arise from losing time in caring, limiting opportunities for personal growth, and education [13]. The stress of caregiving can also have a detrimental effect on the well-being of those receiving it, leading to situations where they are abandoned or institutionalized [13]. Former studies have pointed out various risk factors associated with the caregiving burden, such as female gender [15], low education level [16], co-residence with the care recipient, financial stress, longer caregiving hours [17], lack of choice in being a caregiver, perceived patient distress [13], the closer relationship between caregiver and care recipient (spouses) [18], older age of the caregiver, and medical and psychological status of the care recipient (recipient’s behavior problem, cognitive impairment, and functional disabilities) [19].

Furthermore, the burden of caring for long periods (treatment for 9 or more months in TB) and diminished QoL has a detrimental impact on the quality of care provided to the recipients. Lyu et al., [20] reported that informal care doesn’t satisfy the British patients’ demand and they prefer formal care for better service and less impact of caring on physical and mental health, which caregiver stress brings to their families. Likewise, in TB, African mothers of children with TB recorded physical and psychological influences of caring and diagnosing by acute and chronic illnesses that led, in some cases, to hospitalization and loss of their ability to provide care to their children [21, 22]. Most studies focus on the QoL of patients with TB, however, a critical gap exists in the literature regarding the QoL of the caregivers of patients with TB. This study hypothesizes that caregivers of patients with TB are a neglected group who experience significantly lower QoL scores compared to the general population. So, this survey aimed to assess the domains of QoL of TB caregivers and to estimate their possible determinants in Alexandria, Egypt.

## Methodology

### Study design and sampling techniques

**Fig. 1:**
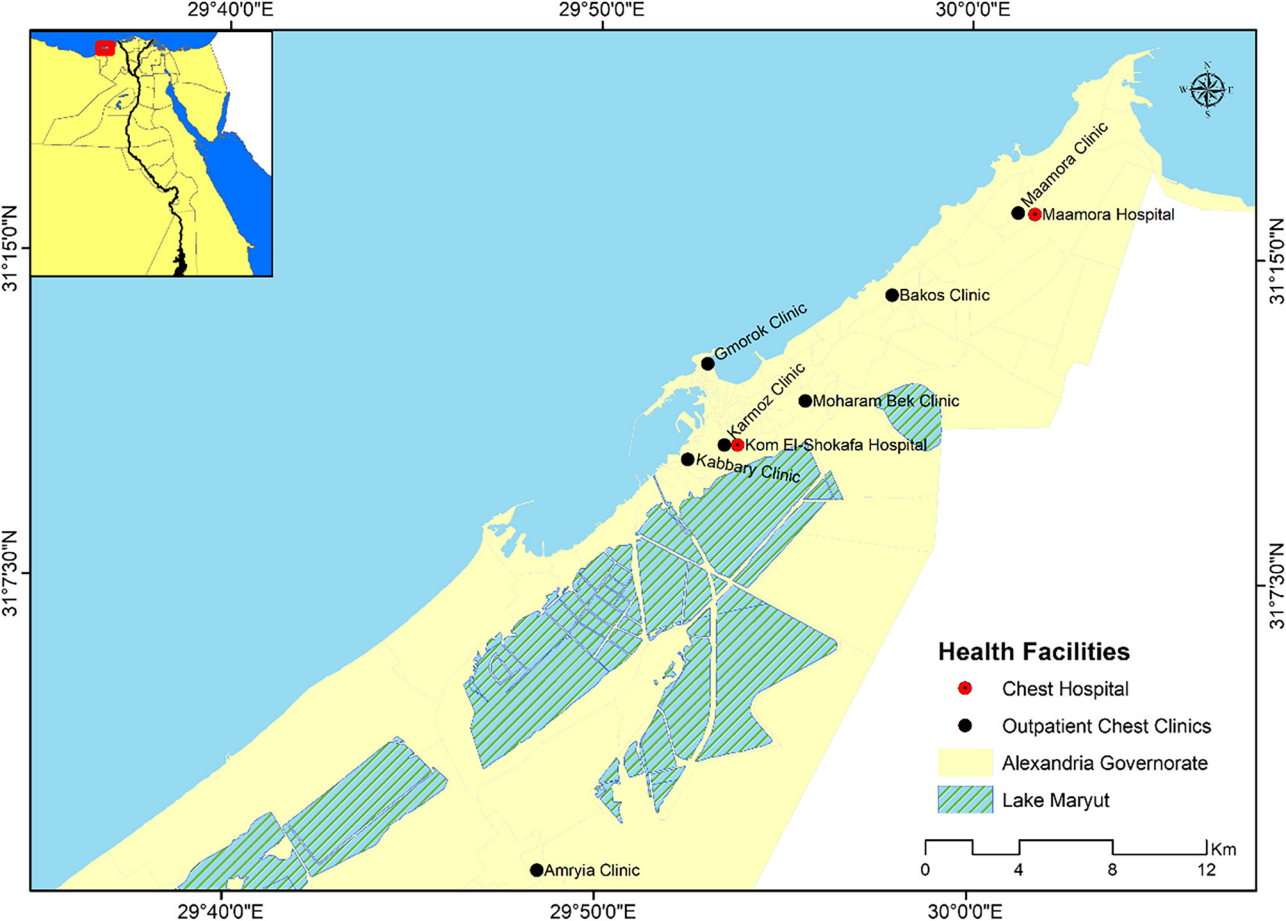
Distribution of chest healthcare facilities in Alexandria, Egypt

A cross-sectional study was conducted at chest clinics affiliated with the MoHP in Alexandria. The city has nine government health facilities dedicated to chest care, including seven outpatient chest clinics—Maamora, Bakos, Gmorok, Amryia, Karmoz, Kom El-Shokafa, and Kabbary—and two chest hospitals located in Maamora and Kom El-Shokafa, which serve as isolation centers for patients with multidrug-resistant tuberculosis (MDR-TB). Figure 1.

We considered the cluster sampling technique to keep the precision and representation of the sample. After calculating the sample size and counting all the clusters, we listed the diagnostic and treatment units (DTU) for each region (administrative zone). Then, we developed a random cluster sampling technique in which hospitals and chest clinics were randomly picked. Ultimately, we chose five facilities—the Maamora Chest Clinic, Bakos Chest Clinic, Amryia Chest Clinic, Gmorok Chest Clinic and Kabbary Chest Clinic—to participate in the study. The study participants were recruited consecutively till the desired sample size was reached.

## Study participants

### Inclusion and exclusion criteria

We recruited caregivers aged ≥ 18 years who assumed primary responsibility for caring for a patient of their relatives with pulmonary or extra-pulmonary TB at both treatment phases (intensive and continuation). We excluded caregivers who were already receiving therapeutic interventions to decrease their burden, as well as individuals who aided those who lived in nursing homes or hospitalized patients, and caregivers who refused to participate.

### Sample size

Hawthorne et al. [23] reported a mean World Health Organization Quality of Life– BREF (WHOQOL-BREF) score of 73.5 ± 18.1 in the general population. Based on a pilot study, the mean score for TB caregivers is expected to be 15% lower (estimated at 62.5) than the general population, with an assumed identical standard deviation (SD) = 18.1. Using G*Power statistical software (version 3.1.9.6), a sample size of 144 TB caregivers was determined to achieve approximately 95% power with a two-tailed analysis (α = 0.05).

### Data collection

We conducted structured face-to-face interviews from May 1st, 2023, till the end of September 2023, at the chest clinics to collect data using a structured questionnaire. Before data collection, the interviewers were trained about TB and infection control measures in the chest clinics. They conducted the interviews in separate ventilated spaces and wore an N95 respirator mask in a medium similar to that previously published research conducted on TB patients in the same settings [24]. The questionnaire consisted of four sections; the first section was completed from the patient’s profiles medical data; type of TB (pulmonary vs. extrapulmonary), treatment phase (intensive vs. continuation phase), and resistance to treatment (MDR vs. (drug sensitive (DS)). The second section was personal and demographic data of the patient’s caregiver, including age, sex, education level, marital status, occupation, degree of relativity, history of chronic diseases, and whether he/she afford the burden of caregiving alone. The third section included socioeconomic level-related questions such as mother’s education and employment, father’s education and employment, computer use, income per capita, family size, crowding index, sewage disposal, and refuse-disposal [25]. Based on these variables, participants were classified into low (scored < 40.0%), middle (scored 40.0% to < 70.0%), or high income (scored ≥ 70.0%) [25]. The fourth section utilized the Arabic-validated form of the WHOQOL-BREF instrument (S3) [26, 27] across four domains: physical, social, psychological, and environmental [28].

The WHOQOL-BREF comprised 26 items, the first two items for evaluating general QoL and general health, and the rest 24 items for assessing QoL in four main domains: physical domain (seven items), psychological domain (six items), social relationship domain (three items), and environmental domain (eight items). The tool follows a scoring system, where each question is rated on a 5-point Likert scale, ranging from 1 (very poor/very dissatisfied/none/never) to 5 (very good/very satisfied/extremely/always). Then, the scores of all four domains were summed and scaled positively, transformed to a 0–100 scale with higher scores indicating better QoL. The estimated acceptable values of the QoL domains in the general population are as follows: physical health QoL = 73.5 ± 18.1, psychological QoL = 70.6 ± 14.0, social relationship QoL = 71.5 ± 18.2, and environmental QoL = 75.1 ± 13.0. Respondents whose scores were above these thresholds were classified as having good quality, while those with scores below the thresholds were classified as having poor quality.

### Ethical approval and consent to participate

Ethical review and approval of the study protocol was obtained by the Ethical Committee of the Central Directorate for Research and Health Development. REC (Com. No/Dec. No: 2-3-2023/11) Informed consent was obtained from all the study participants and legal representatives for illiterates and minors.

### Statistical analysis

Descriptive analysis was employed to outline the demographic characteristics of the participants. Categorical variables were expressed as frequencies (percentages), while continuous variables were presented as means ± SD. The assumptions of normal distribution were assessed through the test of normality and visual examination of histograms. Differences between QoL domains were investigated using the independent t-test or ‎One-way ANOVA.‎ We compared the findings of our sample with those reported in recent studies involving Egyptian TB patients [24], non-TB general population [29], and global reference populations [23] and compared its statistical significance using the independent t-test. Additionally, multivariate linear regression model was utilized to identify the predictors of the domains of QoL among caregivers of TB patients. Each WHOQOL-BREF domain (physical, psychological, social relations, and environment) was analyzed as a separate dependent variable. The model accounted for the potential interrelationships between these dependent factors. The significance level was set at *p* < 0.05. All statistical analyses were conducted using R version 4.2.1.

## Results

**Fig. 2:**
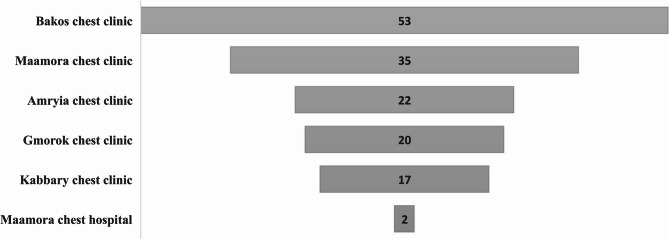
Spatial distribution of study participants

The study included a total of 149 participants recruited from six locations across the Alexandria governorate, as illustrated in Fig. 2. The response rate exceeded 95% as only 4 caregivers declined to participate in the survey. Females constituted 83.9% of the cohort. Of the participants, 76% were married, close to 70% were aged between 35 and 65 years, exhibiting a mean age of 45.2 ± 13.8 years, 49.7% reported no comorbidities, 88.6%, resided in urban areas, 38.3% of the participants were illiterate, 81.2% were 1st-degree relatives. Among the patients, 36.4% were identified as illiterate, 71.1% were diagnosed with pulmonary TB, while 39.6% were in the intensive phase of treatment, and 1.5% exhibited MDR-TB. Table 1. The distribution of socioeconomic levels, calculated using the Fahmy et al. (2015) scale, showed that 67.1% of participants fell within the intermediate class. Financially, 36.2% reported inadequate income. Full socioeconomic assessment details are provided in Supplementary Table S1.

**Table 1:** Sociodemographic and health-related profile of TB patients and their caregivers

Variables (*N* = 149)	*n* (%)
**Patients’ characteristics**	
TB site, pulmonary	106 (71.1%)
Treatment phase, intensive phase	59 (39.6%)
MDR-TB, yes	2 (1.3%)
Educational level- patient	
• Illiterate/read and write	54 (36.2%)
• Basic education level	44 (29.5%)
• High school or above	51 (34.3%)
**Caregivers’ characteristics**	
Gender, female	125 (83.9%)
Age group (in years)	
• 18–34	32 (21.5%)
• 35–49	58 (38.9%)
• 50–64	46 (30.9%)
• ≥ 65 years	13 (8.7%)
Age years, mean ± SD	45.2 ± 13.8
Age years (min, max)	(18.0, 76.0)
Marital status	
• Married	114 (76.5%)
• Single	14 (9.4%)
• Widow/Divorced	21 (14.1%)
Live in an urban area	132 (88.6%)
Educational level- caregiver	
• Illiterate/read and write	57 (38.3%)
• Basic education level	43 (28.8%)
• High school or above	49 (32.9%)
Relation to patient	
• First degree	121 (81.2%)
• Other	28 (18.8%)
Other collaborators in the care tasks, yes	69 (46.3%)
Chronic diseases	
• No	74 (49.7%)
• One co-morbidity	43 (28.8%)
• More than one co-morbidity	32 (21.5%)
Socioeconomic level	
• Low	49 (32.9%)
• Intermediate	100 (67.1%)

First degree relationes: Intensive phase (the first 2 months of treatment after confirmed diagnosis) or ‎continuation phase, MDR-TB: Multidrug-resistant TB.‎s to patients were either mother, father, son, daughter, wife, or husband. ‎Socioeconomic level was calculated according to Fahmy et al. 2015 score. Detailed ‎questions are present in the supplementary table S1. TB site: either pulmonary or extrapulmonary, ‎Treatment phas

Table 2 presents the scores for the four domains, along with the general health score and the general QoL score for TB caregivers, TB patients, non-TB population, and general population cut-off points. Both TB caregivers and patients showed significantly lower scores than the non-TB and general populations across all domains. Caregivers consistently reported the lowest scores, particularly in the social (39.7 ± 20.2) and environmental (42.8 ± 17.3) domains, compared to general population benchmarks (71.5 ± 18.2 and 75.1 ± 13.0, respectively). While caregivers and TB patients had comparable scores in most domains, TB patients reported significantly better social QoL (50.3 ± 20.6 vs. 39.7 ± 20.2; *p* < 0.001). Caregivers also had poorest general health.

**Table 2:** Comparison of QoL scores among TB caregivers, TB patients, Non-TB population, and general population cut-off points

Domain	Caregivers (*N* = 149)	TB patients	Non-TB population	The general population (Cut-off points)
Physical, Mean ± SD	44.8 ± 18.4	42.4 ± 17.8 (*p* = 0.231)	68.3 ± 16.9 (*p* < 0.001)	73.5 ± 18.1
Phycological, Mean ± SD	42.0 ± 17.4	41.9 ± 15.1 (*p* = 0.956)	60.9 ± 13.7 (*p* < 0.001)	70.6 ± 14.0
Social relations, Mean ± SD	39.7 ± 20.2	50.3 ± 20.6 (*p* < 0.001)	56.5 ± 24.1 (*p* < 0.001)	71.5 ± 18.2
Environmental, Mean ± SD	42.8 ± 17.3	44.5 ± 12.8 (*p* = 0.307)	57.1 ± 19.1 (*p* < 0.001)	75.1 ± 13.0
General health, Mean ± SD	2.1 ± 1.0	3.0(2.0–4.0)[Median (IQR)]	3.6 ± 0.9	-
General QoL, Mean ± SD	2.9 ± 1.0	2.0 (2.0–3.0) [Median (IQR)]	3.7 ± 1.0	-

Differences in quality of life domains between caregivers, TB patients (*N* = 180), and individuals without TB (*N* = 2008) were statistically assessed using an independent samples t-test at a significance level of 0.05

Through bivariate analysis, notable differences in mean scores within the physical domain were identified based on gender, age group, marital status, and caregiver comorbidities, with *p* = 0.023, 0.001, 0.002, and < 0.001, respectively. In the psychological domain, mean scores exhibited significance to gender, marital status, caregiver comorbidities, and socioeconomic level, yielding p-values of 0.008, 0.029, 0.012, and 0.031, respectively. The social domain demonstrated significance solely in marital status (*p* < 0.001), indicating that married individuals had higher scores. In the environmental domain, gender (*p* = 0.017), educational level (*p* = 0.017), marital status (*p* = 0.022), and socioeconomic level (*p* = 0.006) all showed significant associations, as detailed in Supplementary Table S2.

Table 3 presents the results of the multivariate linear regression models assessing predictors across the four WHOQOL-BREF domains. In the physical domain, older age (≥ 65 years) predicted lower QoL (*β* = −16.45, *p* = 0.022), while male gender and absence of chronic disease were associated with higher scores (*β* = 10.48, *p* = 0.022 and *β* = 15.51, *p* < 0.001, respectively). The model explained 27.78% of the variance (F-statistic = 3.682, df = 14/134, *p* < 0.001). The model showed a reasonable fit (R² = 21.41%; F-statistic = 2.608, df = 14/134, *p* < 0.001). The psychological domain was positively affected by the absence of chronic disease (*β* = 8.23, *p* = 0.015). For social relations, single and widowed/divorced individuals reported markedly lower QoL than married participants (*β* = −20.96, *p* = 0.003 and *β* = −20.18, *p* < 0.001, respectively). The model accounted for 24.30% of the variance (F-statistic = 3.073, df = 14/134, *p* < 0.001). Lastly, in the environmental domain, receiving additional caregiving support correlated with improved QoL (*β* = 5.42, *p* = 0.039). The model explained 21.26% of the variance (F-statistic = 2.585, df = 14/134, *p* = 0.002). The full multivariate models, including all predictors for each QoL domain, are presented in Supplementary Table S3.

The intercept values in the regression models reflect the estimated mean domain scores for the reference group, defined as participants aged 18–34, female, living in rural areas, married, with an illiterate caregiver, with more than one co-morbidity, in the continuation treatment phase, not receiving additional caregiving help, and of intermediate socioeconomic status. Notably, these interceptions varied across domains: Social relations had the highest baseline score (49.89), followed by physical (43.05), then environment (33.34), and psychological had the lowest baseline score (31.95).

**Table 3:** Multivariate linear regression analysis of predictors across the four WHOQOL-BREF domains

Model	Predictor	Estimate (β)	SE	*p*-Value
**Physical**	Intercept	43.05	7.74	< 0.001
	Age group (65–76)	−16.45	7.14	0.022
	Gender: Male	10.48	4.51	0.022
	No chronic disease	15.51	4.20	< 0.001
**Psychological**	Intercept	31.95	6.14	< 0.001
	Gender: Male	7.06	3.57	0.0500
	No chronic disease	8.23	3.33	0.015
**Social Relations**	Intercept	49.89	8.30	< 0.001
	Marital status: Single	−20.96	7.02	0.003
	Marital status: Widow/Divorced	−20.18	4.87	< 0.001
**Environment**	Intercept	33.34	6.42	0.002
	Other help in patient care: Yes	5.42	2.61	0.039

## Discussion

TB disease is a public health problem that can hinder the life goals of patients to different extents. Families of the patients have a crucial role in supporting them to overcome their health issues during and after the treatment journey [30]. This multicenter, cross-sectional study using the Arabic-validated form of the WHOQOL-BREF questionnaire assessed the impact of TB on the QoL of the caregivers of people with this disease.

### Findings of the study

Most of the caregivers in the current study were females, first-degree relatives of the patients. These results support previous research into this brain area, which links caregiving burden and gender. A study conducted in Saudi Arabia reported that more than three-quarters of the caregivers of patients on hemodialysis were their mothers, daughters, and wives [31]. Other studies conducted in Egypt and Lebanon indicated similar results [32, 33]. These results could be interpreted by the social norm in Middle Eastern societies that influences women to take on more caregiving responsibilities without sufficient support, and precipitates lower scores in social, psychological, and environmental domains for them in the current study, as well as in the former studies [31, 34].

The current study reported comparative scores between the caregivers of patients with TB and those reported by Hammouda et al., [24] about QoL of TB patients in a similar society in all domains except the social and general health domains, which have significantly worse scores. However, Itagi et al., [35] reported lower scores of quality of sleep in TB patients than their family caregivers without clear reasons. Compared to the shortcuts of the general populations [23, 29], caregivers have 30.0–50.0% lower scores in all domains of QoL. Likewise, Fana et al., [30] reported in their qualitative study that caregivers of TB experienced social exclusion, anxiety, and fears of losing love and social support, besides their fears of infection transmission. This could be attributed to the social isolation of caregivers and withdrawal from their routines and social habits to focus on caring for their patients [36]. An additional possible determinant for lowering the social domain scores may be social stigma and discrimination, which many caregivers share with their patients due to TB. The results of the current study were consistent with several studies that reported lower social scores due to fears of labeling their family members with socially stigmatizing diseases. In our sample, the majority of caregivers were female, aligning with existing literature that shows women tend to assume greater caregiving responsibilities and invest more time in such roles than men. These results complement our earlier study, which found that TB patients—mostly male—had significantly lower social QoL scores than non-TB individuals. Notably, caregivers in the current study reported lower social domain scores than the TB patients themselves. This may have a consequential effect on their caregiving responsibilities [37–41] and the economic burden of the disease [24, 42].

Regarding QoL domain scores, both reference groups (TB patients and non-TB population) assessed the QoL domains two years after the COVID-19 pandemic, which was a major contributing factor to the overall decline in QoL during that period. Despite subsequent societal recovery, the disparity in QoL scores between caregivers and the general population remains substantial.

### Factors associated with QoL domains of caregivers

#### The physical and psychological domains

Male caregivers reported significantly better scores in physical and psychological QoL domains compared to females. This could be interpreted by the differences in the coping mechanisms between males and females, whereas males focus on problem-solving mechanisms in stressful conditions, while females depend on changing their emotional responses to stressful conditions [43]. Furthermore, females are more vulnerable to developing mental health disorders, including depression and anxiety, due to hormonal, genetic, and neurobiological influences [44]. While caregivers residing in urban areas tended to have higher physical domain scores than their rural counterparts, this difference did not reach statistical significance, though the trend may still be of practical importance. These findings agreed with those obtained by Galal et al., [45] who reported fewer health complaints in urban than rural areas in Egypt. Moreover, the absence of chronic diseases was associated with higher scores of physical and psychological domains. This finding could be interpreted by lower capacity for movement and practicing normal life for caregivers who suffer from chronic diseases compared to healthy ones. Likewise, Megari [46] indicated that the presence of chronic diseases hinders the overall health of patients by constraining their ability to lead fulfilling lives, restricting their functional capacity, and productivity. Qadire et al., [47] reported a lower QoL in populations with multiple chronic diseases than those with just one chronic condition.

#### The social domain

Unmarried caregivers (single, widowed, or divorced) were associated with lower scores. This finding seems to be consistent with other research conducted by Arasu et al., [48] which reported higher social score domains in married caregivers of children with disabilities. Similarly, Kim et al., [49] reported comparable results among female caregivers in the Korean society. It seems possible that these results are due to the emotional support that spouses offer to their partners. This could mitigate the stress and anxiety of care.

#### The environmental domain

Male caregivers demonstrated higher scores compared to females; however, this association was only marginally significant, suggesting a potential trend that warrants further exploration. Additionally, Caregivers who received help from others in providing care had significantly higher environmental domain scores, reflecting the benefits of shared responsibilities in reducing burden and enhancing well-being. Consistent with findings by Leng et al., [50], social support is strongly correlated with caregiver QoL. Encouraging caregivers, particularly those unemployed or long-term carers, to seek assistance from family and friends, alongside mental health education and support network strengthening, may effectively improve their QoL [50].

Finally, the relatively high intercept values across all QoL domains—representing baseline scores for younger, female, rural, married caregivers with low education, multiple comorbidities, no assistance, and in the continuation treatment phase—suggest that even in the absence of favorable predictors, caregivers maintained moderate QoL levels, potentially reflecting underlying resilience or culturally embedded caregiving norms in the Egyptian context.

#### Implications of the study

Assessing the QoL of caregivers of patients with TB points out key areas of strain, such as stigma, social support, and mental health challenges, providing valuable insights for policymakers to design targeted interventions. Assessing the socioeconomic status of caregivers endorses effective resource allocation, as studies show that those from lower socioeconomic backgrounds often report lower QoL scores. By implementing tailored support programs, such as psychosocial counseling, financial assistance, and community-based services, the well-being and health outcomes of caregivers can be significantly improved. Consequently, this enhances the performance of caregiving, patient adherence to treatment, and recovery, leading to strengthening the overall effectiveness of TB control programs.

#### Strengths and limitations

This study includes various strengths that contribute to credibility and reliability. First, to the best of our knowledge, this could be the first quantitative study that addressed the QoL among caregivers of adult patients with TB in the region. Second, we collected participants from different settings representing the different criteria for caregivers (different degrees of relativity, different residency, and different educational levels) in Alexandria. However, the study had many limitations, including the inherent limitations of the adopted study design (cross-sectional survey), such as recall bias, inability to investigate causality, and difficulty assessing QoL at different treatment stages. Second, we couldn’t assess QoL on a larger scale in different governments. Finally, the inability to adequately include caregivers of MDR-TB patients in the analysis, as only two such cases were identified. These patients were hospitalized for extended periods, making it difficult to reach their caregivers; thus, MDR-TB status could not be included as a factor in the regression model.

## Conclusions

In conclusion, this study highlighted the care burden of the caregivers of patients with TB and reported diminished scores in all QoL domains. Older age notably reduced physical QoL. The male gender and absence of chronic diseases improved both physical and psychological domain scores. Socially, unmarried (single/widowed/divorced) caregivers reported substantially lower QoL than married individuals. Environmental QoL improved with additional caregiving support. These findings underscore the crucial need for strategies targeting improving the QoL of caregivers as part of patient care programs. Empowering caregivers could assist the community by spreading awareness, encouraging the population at risk for screening and applying prevention measures, reducing the stigma of TB, and decreasing the burden on the healthcare systems.

## Supplementary Information

Supplementary Material 1

Supplementary Material 2

## Data Availability

The data sets used and analyzed during the current study are available by emailing the corresponding author upon reasonable request.

## Abbreviations

CDC: The Centers for Disease Control and Prevention ‎
DOTS: Directly observed therapy short-course system
DS: Drug sensitive
DTU: Diagnostic and treatment units
IDSA: Infectious Diseases Society of America
LMICs: Low and middle-income countries
MDR: Multidrug resistant
MoHP: Ministry of Health and Population
QoL: Quality of life
TB: Tuberculosis
WHOQOL-BREF: World Health Organization Quality of Life Brief Version questionnaire
