# Supplementary material for: Exploring the quality of life and its determinants among caregivers of patients with tuberculosis: a cross-sectional study
Source: BMC Public Health. 2025 Aug 19;25:2832. doi: 10.1186/s12889-025-23854-2 (PMC12362928; doi:10.1186/s12889-025-23854-2)
Supplement: Supplementary file 2 — Supplementary Material 2 [file 12889_2025_23854_MOESM2_ESM.docx]

**Table S1:** Socioeconomic level determinants questions.

| **Variables (N =149)** | **n (%)** |
| --- | --- |
| **Educational level of main female of family** | |
| Illiterate/ read and write | 67 (45.0%) |
| Literate certificate/Primary | 24 (16.1%) |
| Preparatory | 15 (10.1%) |
| Secondary | 39 (26.2%) |
| University | 4 (2.7%) |
| **Educational level- father of family** | |
| Illiterate/ read and write | 61 (40.9%) |
| Literate certificate/ Primary | 27 (18.1%) |
| Preparatory | 31 (20.8%) |
| Secondary | 27 (18.1%) |
| University | 3 (2.0%) |
| **Dose mother work, yes** | 28 (18.8%) |
| **Dose father work, yes** | 85 (57.0%) |
| **Using computer in house** |  |
| Never | 128 (85.9%) |
| Sometimes | 11 (7.4%) |
| Plenty of times | 10 (6.7%) |
| **Per-capita household income** |  |
| Enough only | 33 (22.1%) |
| Not enough + small loan | 54 (36.2%) |
| Not enough + big loan | 23 (15.4%) |
| Not enough + loan not repaid | 39 (26.2%) |
| **Family number** |  |
| Less than 5 | 78 (52.3%) |
| 5 to 7 | 56 (37.6%) |
| More than 7 | 15 (10.1%) |
| **Crowding index, Median (IQR)** | 2.0 (2.0, 2.0) |
| **Sewage network, yes** | 144 (96.6%) |
| **Sewage disposal by caregiver, yes** | 130 (87.2%) |
|  |  |

**Table S2:** Sociodemographic characteristics and mean scores (SD) of qualities of life domains among caregivers of tuberculosis (TB) Patients

| **Domain** | **Gender**  **Statistical test: independent t test** | | | **Age groups**  **Statistical test: one way AVOVA** | | | | | **caregiver educational level**  **Statistical test: one way AVOVA** | | | | **Marital status**  **Statistical test: one way AVOVA** | | | |
| --- | --- | --- | --- | --- | --- | --- | --- | --- | --- | --- | --- | --- | --- | --- | --- | --- |
|  | **Female** | **Male** | **p value** | **18 -<35** | **35 -< 50** | **50-<65** | **65-76** | **p value** | **Primary** | **Secondary** | **University** | **p value** | **Married** | **Single** | **Widow/ Divorced** | **p value** |
| **Physical Qol** | 55.1 (19.0) | 65.0 (21.1) | **0.023** | 67.3 (15.3) | 55.8 (17.4) | 54.0 (22.3) | 56.7 (19.6) | **0.001** | 56.1 (18.0) | 58.0 (20.2) | 65.8 (12.5) | **0.588** | 55.4 (19.8) | 73.7 (11.1) | 52.7 (18.0) | **0.002** |
| **Physcological Qol** | 47.8 (14.2) | 56.6 (16.3) | **0.008** | 51.7 (16.0) | 47.7 (12.0) | 51.5 (16.1) | 49.2 (14.9) | **0.127** | 52.5 (14.4) | 52.2 (15.4) | 54.2 (13.2) | **0.063** | 48.1 (14.4) | 59.2 (14.4) | 49.0 (15.7) | **0.029** |
| **Social.relations Qol** | 48.1 (20.3) | 54.2 (21.1) | **0.188** | 45.6 (18.8) | 50.9 (21.3) | 52.0 (20.2) | 49.1 (20.5) | **0.172** | 54.6 (20.0) | 53.8 (21.4) | 39.3 (22.4) | **0.134** | 53.7 (20.3) | 37.5 (12.1) | 31.7 (12.3) | **< 0.001** |
| **Enviromental Qol** | 45.1 (15.3) | 53.4 (15.3) | **0.017** | 49.7 (13.0) | 43.9 (14.3) | 48.5 (17.8) | 46.5 (15.5) | **0.209** | 49.7 (13.7) | 50.1 (16.7) | 52.2 (8.4) | **0.017** | 44.8 (16.3) | 56.5 (9.0) | 48.7 (11.9) | **0.022** |
| **Overall Qol** | 2.4 (0.9) | 2.6 (1.0) | **0.313** | 2.9 (0.7) | 2.4 (0.9) | 2.3 (1.0) | 2.4 (0.9) | **0.018** | 2.1 (0.9) | 2.7 (0.9) | 3.3 (0.8) | **0.013** | 2.4 (0.9) | 3.2 (0.7) | 2.3 (1.0) | **0.004** |
| **overall satisfaction** | 3.4 (0.9) | 3.5 (0.8) | **0.489** | 3.8 (0.7) | 3.4 (0.9) | 3.1 (0.9) | 3.4 (0.9) | **< 0.001** | 3.0 (0.9) | 3.5 (0.9) | 4.3 (0.5) | **0.028** | 3.3 (0.9) | 4.2 (0.6) | 3.0 (0.9) | **< 0.001** |

|  | **Region**  **Statistical test: independent t test** | | | **Relative degree to patient**  **Statistical test: one way AVOVA** | | | | | **Caregiver co morbidities**  **Statistical test: one way AVOVA** | | | | **Socioeconomic level**  **Statistical test: independent t test** | | |
| --- | --- | --- | --- | --- | --- | --- | --- | --- | --- | --- | --- | --- | --- | --- | --- |
|  | **Rural** | **Urban** | **p value** | **1st** | **2nd** | **3rd** | **4th** | **p value** | **No** | **One Co-morbidity** | **More than one co-morbidity** | **p value** | **Intermediate** | **Low** | **p value** |
| **Physical Qol** | 48.7 (23.4) | 57.8 (18.9) | **0.074** | 56.1 (20.4) | 57.1 (16.9) | 64.8 (13.9) | 60.7 (12.4) | **0.7** | 64.4 (17.3) | 52.2 (18.5) | 45.1 (18.9) | **< 0.001** | 58.9 (20.2) | 52.3 (17.7) | **0.051** |
| **Physcological Qol** | 45.8 (16.5) | 49.7 (14.7) | **0.317** | 48.9 (15.4) | 49.3 (13.6) | 54.8 (11.1) | 51.4 (12.7) | **0.779** | 52.5 (13.9) | 47.9 (15.4) | 43.5 (14.8) | **0.012** | 51.1 (15.7) | 45.5 (12.3) | **0.031** |
| **Social.relations Qol** | 51.5 (22.3) | 48.8 (20.3) | **0.615** | 49.1 (20.9) | 50.0 (20.4) | 47.6 (18.5) | 47.2 (17.3) | **0.992** | 46.5 (20.5) | 53.9 (21.1) | 48.7 (19.2) | **0.172** | 50.5 (19.7) | 46.3 (22.1) | **0.237** |
| **Enviromental Qol** | 41.0 (19.1) | 47.2 (15.0) | **0.124** | 45.7 (15.8) | 48.1 (13.4) | 53.6 (16.5) | 49.0 (17.2) | **0.576** | 48.9 (14.3) | 44.6 (16.7) | 43.4 (16.3) | **0.163** | 48.9 (15.5) | 41.5 (14.7) | **0.006** |
| **Overall Qol** | 2.6 (1.0) | 2.4 (0.9) | **0.514** | 2.4 (0.9) | 2.9 (0.8) | 2.6 (1.0) | 2.7 (0.6) | **0.089** | 2.7 (0.9) | 2.3 (0.9) | 2.1 (1.0) | **0.005** | 2.6 (0.9) | 2.2 (1.0) | **0.008** |
| **overall satisfaction** | 2.9 (0.7) | 3.4 (0.9) | **0.032** | 3.4 (0.9) | 3.5 (0.7) | 3.1 (0.9) | 3.7 (0.6) | **0.779** | 3.6 (0.8) | 3.4 (0.9) | 2.8 (0.9) | **< 0.001** | 3.5 (0.9) | 3.2 (0.8) | **0.038** |

|  | **Treatment phase**  **Statistical test: independent t test** | | | **Disease position**  **Statistical test: independent t test** | | | **Patient educational level**  **Statistical test: one way AVOVA** | | | | | |
| --- | --- | --- | --- | --- | --- | --- | --- | --- | --- | --- | --- | --- |
|  | **continuation** | **intensive** | **p value** | **Extra-pulmonary** | **Pulmonary** | **p value** | **Illiterate/ read and write** | **Literate certificate/ Primary** | **Preparatory** | **Secondary** | **University** | **p value** |
| **Physical Qol** | 55.8 (20.5) | 58.1 (18.1) | **0.489** | 59.1 (16.3) | 55.8 (20.8) | **0.359** | 55.9 (20.9) | 57.7 (16.1) | 61.2 (17.8) | 56.1 (21.1) | 52.4 (18.5) | **0.748** |
| **Physcological Qol** | 48.3 (14.9) | 50.7 (14.8) | **0.333** | 50.5 (14.8) | 48.7 (15.0) | **0.519** | 45.3 (13.8) | 53.8 (11.7) | 52.4 (17.4) | 51.6 (15.3) | 45.5 (15.0) | **0.072** |
| **Social.relations Qol** | 50.1 (20.6) | 47.6 (20.4) | **0.47** | 51.2 (21.0) | 48.3 (20.3) | **0.437** | 46.3 (18.6) | 56.7 (16.8) | 56.9 (26.2) | 46.8 (20.6) | 41.0 (16.5) | **0.045** |
| **Enviromental Qol** | 47.1 (15.8) | 45.5 (15.3) | **0.544** | 46.2 (15.8) | 46.6 (15.5) | **0.907** | 42.8 (13.3) | 49.9 (12.8) | 52.3 (19.1) | 47.0 (17.3) | 44.3 (13.2) | **0.106** |
| **Overall Qol** | 2.4 (0.9) | 2.5 (1.0) | **0.657** | 2.2 (0.9) | 2.5 (0.9) | **0.068** | 2.3 (0.9) | 2.2 (0.9) | 2.7 (0.9) | 2.6 (0.9) | 2.5 (1.2) | **0.301** |
| **overall satisfaction** | 3.3 (0.9) | 3.4 (0.9) | **0.527** | 3.4 (0.8) | 3.4 (1.0) | **0.758** | 3.4 (0.9) | 3.2 (0.9) | 3.5 (0.8) | 3.3 (0.9) | 3.7 (1.0) | **0.607** |

**Table S3:** Multivariate regression estimates across quality-of-life domains

| **Predictor** | **Physical**  β (p-value) | **Psychological**  β (p-value) | **Social relations**  β (p-value) | **Environment**  β (p-value) |
| --- | --- | --- | --- | --- |
| Intercept | 43.05* (p <0.001) | 31.95* (p <0.001) | 49.89* (p <0.001) | 33.34* (p <0.001) |
| Age group (18 to <35) | Ref | Ref | Ref | Ref |
| Age group (35 to <50) | -4.78 (p = 0.310) | 1.15 (p = 0.757) | -1.36 (p = 0.788) | -2.68 (p = 0.493) |
| Age group (50 to <65) | -4.43 (p = 0.395) | 6.25 (p = 0.131) | 2.15 (p = 0.701) | 2.90 (p = 0.502) |
| Age group (65–76) | -16.45* (p =0.022) | -4.11 (p = 0.469) | -3.91 (p = 0.610) | -2.53 (p = 0.670) |
| Gender: Female | Ref | Ref | Ref | Ref |
| Gender: Male | 10.48* (p = 0.022) | 7.06 (p = 0.050) | 7.02 (p = 0.149) | 7.10 (p = 0.059) |
| Region: Rural | Ref | Ref | Ref | Ref |
| Region: Urban | 9.43 (p = 0.058) | 4.46 (p = 0.255) | -0.01 (p = 0.998) | 5.94 (p = 0.148) |
| Marital status: Married | Ref | Ref | Ref | Ref |
| Marital status: Single | 4.18 (p = 0.524) | 3.96 (p = 0.447) | -20.96* (p =0.003) | 1.84 (p = 0.735) |
| Marital status: Widow/Divorced | 2.38 (p = 0.602) | 3.02 (p = 0.402) | -20.18* (p <0.001) | 6.26 (p = 0.0989) |
| Caregiver education: Illiterate | Ref | Ref | Ref | Ref |
| Caregiver education: Primary or preparatory | -4.07 (p = 0.290) | 2.74 (p = 0.368) | 4.26 (p = 0.302) | 4.64 (p = 0.147) |
| Caregiver education: Secondary or above | -4.09 (p = 0.299) | 2.79 (p = 0.370) | 4.41 (p = 0.295) | 5.59 (p = 0.088) |
| No chronic disease | 15.51* (p <0.001) | 8.23 *(p = 0.015) | -2.18 (p = 0.628) | 4.06 (p = 0.245) |
| One Co-morbidity | 6.13 (p = 0.161) | 3.99 (p = 0.249) | 4.63 (p = 0.322) | 0.79 (p = 0.827) |
| More than one Co-morbidity | Ref | Ref | Ref | Ref |
| Treatment phase: Intensive | 2.67 (p = 0.383) | 2.70 (p = 0.266) | 0.37 (p = 0.911) | -1.69 (p = 0.505) |
| Treatment phase: Continuation | Ref | Ref | Ref | Ref |
| Other help in patient care: Yes | 1.70 (p = 0.591) | 4.73 (p = 0.060) | 2.64 (p = 0.435) | 5.42* (p = 0.039) |
| Socioeconomic level: Low | -3.72 (p = 0.285) | -2.49 (p = 0.365) | -3.60 (p = 0.334) | -3.79 (p = 0.189) |
| Socioeconomic level: Intermediate | Ref | Ref | Ref | Ref |
| **Model Statistics**  F- statistic  Degree of freedom  p-value  R^2^ | 3.682  14/ 134  <0.001  27.78% | 2.608  14/ 134  <0.001  21.41% | 3.073  14/ 134  <0.001  24.30% | 2.585  14/ 134  0.002  21.26% |
